# Neutrophil-Lymphocyte Ratio (NLR) Reflects Myocardial Inhomogeneities in Hemodialyzed Patients

**DOI:** 10.1155/2020/6027405

**Published:** 2020-09-03

**Authors:** Kamila Bołtuć, Arkadiusz Bociek, Robert Dziugieł, Martyna Bociek, Tomasz Zapolski, Wojciech Dąbrowski, Andrzej Jaroszyński

**Affiliations:** ^1^Collegium Medicum, Jan Kochanowski University in Kielce, Poland; ^2^Faculty of Medical Science, Higher School of Economy, Law and Medical Science of Professor Edward Lipiński in Kielce, Poland; ^3^Department of Cardiology, Medical University of Lublin, Poland; ^4^Department of Anesthesiology and Intensive Care, Medical University of Lublin, Poland

## Abstract

**Introduction:**

Cardiovascular diseases (CVDs) are a leading cause of death in chronically hemodialyzed (HD) patients. In this group, inflammation exerts significant impact on the prevalence of CVD morbidity and mortality. Spatial QRS-T angle is an independent and strong predictor of CV events, including sudden cardiac death (SCD), both in general population and HD patients. Pathogenesis of widened QRS-T angle is complicated and is not well established.

**Objectives:**

The study is aimed at evaluating whether inflammation process can contribute to the wide QRS-T angle. *Patients and Methods*. The retrospective study was performed on 183 HD patients. The control group consisted of 38 patients. Demographic, biochemical, vectorcardiographic, and echocardiographic data were evaluated in all patients. Inflammation process was expressed as neutrophil-lymphocyte ratio (NLR), as well as C-reactive protein (CRP).

**Results:**

Both NLR (3.40 vs. 1.95 (*p* < 0.0001)) and spatial QRS-T angle (50.76 vs. 93.56 (*p* < 0.001)) were higher in the examined group, compared to the control group. Similarly, CRP was higher in the examined group than in the control group (8.35 vs. 4.06 (*p* < 0.001), respectively). The QRS-T angle correlated with NLR, CRP, some structural echocardiographic parameters, parathormone (PTH), and calcium (Ca) concentrations. Multiple regression analysis showed that NLR is an independent QRS-T angle predictor (*r* = 0.498, *p* = 0.0027). The ROC curve analysis indicated the cut-off point of NLR equaled 4.59, where the sensitivity and specificity were the highest for predicting myocardial inhomogeneities expressed as widened QRS-T angle.

**Conclusion:**

The NLR, as an inflammation marker, may indicate myocardial inhomogeneities in HD patients.

## 1. Introduction

CVD is the most common cause of death in patients chronically receiving HD [1, 2]. Chronic inflammation is highly prevalent in this group and plays a prominent role in CVD morbidity and mortality [1–3]. Moreover, it seems that inflammation has a greater impact for CVD prevalence in HD patients, compared to general population [1, 2].

The spatial QRS-T angle is an independent predictor of CVD risk, including SCD, both in general population and in HD patients. Moreover, it is a death indicator for cardiovascular causes and is also an important factor for general risk of death [4–10]. The spatial QRS-T angle is measured between the vectors of ventricular depolarization and repolarization and it can be determined from standard 12-lead electrocardiogram [6, 9, 11]. Pathogenesis of widened QRS-T angle is complicated and not well established [6]. The studies show that HD patients have abnormally wide QRS-T angle [9, 10]. It is thought that inflammation can play a role in this process [12].

CRP is an acute-phase protein and the most common marker of inflammation. It is produced by macrophages and hepatocytes [13, 14]. However, in recent years, there was an increase of interest in other inflammatory indicators, including NLR [13, 15]. NLR reflects the presence of systemic inflammation [16, 17]. NLR is easy to determine and it can be based on simple morphology. Its major benefit is a possibility for retrospective calculation [18, 19]. NLR is a quotient of an absolute neutrophil count to absolute count of leucocytes [16, 20]. The norm of NLR is still undetermined, but usually NLR ≥ 4.5 stands for increased risk of death [16, 19]. Previous studies demonstrated that higher NLR values are associated with coronary and periaortic calcification in HD patients, which is an important CVD morbidity and mortality risk factor [1].

The study is aimed at evaluating whether the inflammation process can contribute to wide QRS-T angle.

## 2. Patients and Methods

The retrospective study was performed on 183 ESRD patients treated by hemodialysis. The following exclusion criteria were applied: HD treatment for less than 3 months (to include only patients with ESRD), advanced neoplastic diseases reducing life expectancy to below a year, and patients displaying symptoms of acute infections at baseline (to reduce the possible influence of transient factors on the NLR value). Given that estimating the population size meeting the criteria used in our study was impossible, the sample size calculation was not performed. All available HD patients in Lublin were included. The control group included 38 patients with normal GFR values. The groups did not differ in sex and age structure.

Results of routinely performed transthoracic echocardiography and electrocardiography (ECG) (vectorcardiography (VCG)) were used. Patients underwent these examinations according to the recommendations of the American Society of Echocardiography and European Association of Cardiovascular Imaging [21] every 6 months, as described elsewhere [9]. Based on planar measurements, left ventricle stroke volume (SV), cardiac output (CO), stroke index (SI), left ventricle ejection fraction (EF), E/E`, cardiac index (CI), left ventricle mass index (LVMI), left atrial volume index (LAVI), left ventricle mass (LVM), left atrial volume (LAV), left ventricle end-diastolic and end-systolic volumes (LVEDV and LVESV, respectively) were estimated. To calculate the body surface, the Gehan and George formula was used, as described in detail elsewhere [22, 23].

The digital ECG examination records, obtained with use of Cardiax device (IMED Co. Ltd., Budapest, Hungary), were used. Based on the recorded surface 12-lead resting ECG, the vectorcardiographical parameters were estimated by transforming them into Frank's orthogonal leads, according to inverse Dower matrix [24]. Using the Cardiax software, the spatial QRS-T angle was calculated from the maximum QRS and T vectors. Wider than normal QRS-T angle was established as over 116 degrees for females and over 130 degrees for males [25].

Using automated analyzers, the following biochemical parameters were examined: blood morphology, electrolytes, creatinine, Ca, PTH, CRP, and lipid profile. NLR was calculated as quotient of the absolute number of neutrophils to the absolute number of lymphocytes. These tests were carried out no more than a month prior to the echocardiographic and ECG examinations.

The Bioethical Commission at Medical University in Lublin, Poland, approved this study with decision number: KE-0254/74/2016.

The normality of distribution of the variables was checked with the Shapiro-Wilk test. The variables with normal distribution were presented as the mean ± SD, and these which did not fulfill the normality criterion were presented as the median and range. Pearson's correlation test and multivariable logistic regression were used to the statistical analysis. The aim of the multiple regression analysis was to identify independent predictors of QRS-T angle. Due to the most accurate correlation with QRS-T angle in the Pearson test, left ventricle mass index, left atrial volume index, ejection fraction, PTH, NLR, and Ca were chosen to the multiple regression analysis. Due to a correlation with abovementioned variables and weak correlation with QRS-T angle in the Pearson test, other echocardiographic measurements and CRP were excluded. Also, ROC curve analysis was performed in order to determine the sensitivity, specificity, and cut-off point of NLR as a predictor of QRS-T angle. The considered level of significance was established as *p* value < 0.05. The entire analysis was performed with the TIBCO Software Inc. (2017) Statistica (data analysis software system), version 13 (http://statistica.io).

## 3. Results

There were 99 (54.1%) men and 84 (45.9%) women with an average age of 69.4 years. Patients had the following causes of kidney failure: diabetes mellitus (*n* = 82), glomerulonephritis (*n* = 37), hypertensive nephropathy (*n* = 16), polycystic kidney disease (*n* = 6), obstructive nephropathy (*n* = 5), chronic pyelonephritis (*n* = 4), and unknown/unsure (*n* = 32).

The basic biochemical parameters are presented in Table 1. Both the QRS-T angle and NLR were higher in the examined group, compared to the control group (50.76 vs. 93.56 (*p* < 0.001) and 3.40 vs. 1.95 (*p* < 0.0001), respectively). Similarly, CRP was higher in the examined group than in the control group (8.35 vs. 4.06 (*p* < 0.001), respectively).

Out of the whole group of the examined patients, 82 suffered from diabetes, 124 from hypertension, and 37 used nicotine. The following pharmacological treatment was used: ACE-I/ARB (*n* = 131), *β*-blockers (*n* = 125), statins (*n* = 139), and diuretics (*n* = 43).

The spatial QRS-T angle correlated with LVMI, LAVI, E/E`, EF, QRS duration, QTc, NLR, PTH, Ca, and CRP. The correlations of the abovementioned parameters with QRS-T are presented in Table 2.

Multiple regression analysis (*r* = 0.81, *p* < 0.0001) showed that NLR, but not CRP, was an independent indicator of the QRS-T angle (*p* = 0.0027). The details of the abovementioned analysis are shown in Table 3.

**Figure 1:**
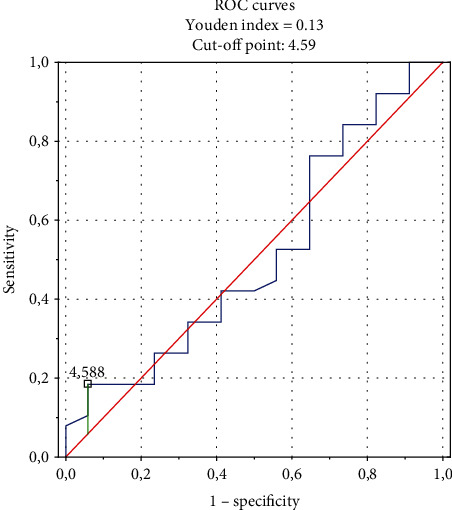
The ROC curves present sensitivity and specificity of NLR in predicting the spatial QRS-T angle.

The ROC curves of NLR as predictor of the QRS-T spatial angle were performed. The analysis showed the AUC equal 0.601 and the sensitivity/specificity equal 0.677/0.601. The cut-off point was 4.59, so the value of NLR equal 4.59 has the biggest sensitivity and specificity. The ROC curves are presented in Figure 1 and described in details in Table 4.

## 4. Discussion

To our knowledge, this is the first study that shows the relation between inflammation process, expressed as NLR, and QRS-T angle, which is considered the indicator of myocardial inhomogeneities. Moreover, our study revealed that NLR is a better marker than CRP in predicting the widening of the spatial QRS-T angle.

Cardiovascular diseases are very common in HD patients, and SCD, especially, is the main cause of death [26]. Additionally, in HD patients, compared to the healthy controls, abnormally wide spatial QRS-T angle was observed, which affected mortality as well. Pathomechanisms between CVD and chronic kidney disease (CKD) are complicated, multifactorial, and yet not completely understood. Various processes, such as vascular calcification [1], endothelial dysfunction [27], and chronic inflammation, among others, significantly contribute to development of these diseases [28]. There is a variety of inflammatory markers, such as CRP [29]; however, they are expensive and imprecise. It is difficult to obtain other inflammatory markers, like interleukin 2 (IL-2) [30] and interleukin 6 (IL-6) [6], in clinical practice, even though they might be more accurate [31]. Moreover, measuring the most common biomarker, which is CRP, with basic technique may not detect low-grade inflammatory process in patients with CKD [29]. It may explain why CRP does not correlate with the QRS-T angle. Thus, there is a need to find a simple, inexpensive, and precise marker, which can estimate the risk of CVD and SCD, especially in CKD patients treated with hemodialysis. Our study confirms that NLR can be that.

Spatial QRS-T angle is perceived as a measure of a global myocardial inhomogeneities [32]. It is a significant and independent predictor of CVD and all-cause mortality [5–8, 11, 33–37]. The results reported by Yamazaki et al. revealed that the QRS-T angle, contrary to classical cardiovascular and ECG risk factors, is the strongest marker of the increased risk of fatal cardiac incidents, including SCD [11]. Its border values differ depending on the sex, but due to different marking methods, they are not strictly standardised [9]. It is thought that abnormal spatial QRS-T angle is defined as ≥116 degrees in females and ≥130 degrees in males [6, 10, 25]. In our study, we have demonstrated that HD patients, but not healthy subjects, had increased values of the QRS-T angle. Owing to the fact that widend QRS-T angle pathogenesis in this group of patients has not been extensively investigated, there is little knowledge about it. However, it is believed that inflammatory process plays an important role in the widening of the spatial QRS-T angle and, consequently, SCD occurance in HD patients. Therefore, NLR, with the highest sensitivity and specificity among other inflammatory biomarkers, seems to be the most precise.

NLR, as a commonly available marker of inflammation, has a great prognostic value for many different conditions and diseases [1, 2, 16, 29, 38–44], including cardiovascular disorders [20, 40, 42, 45]. Previously published studies have revealed that increased NLR values are observed in patients with hypertension [45], aneurysms in ascending aorta [20], and stable coronary artery disease [42]. According to the study performed by Benites-Zapata et al., high NLR levels were associated with increased mortality in patients with acute heart failure and increased heart transplantation risk [46]. Chronic obstructive pulmonary disease (COPD) [43], psoriasis [38], vitamin D deficiency [41], which is observed in the early stages of CKD [47], and also Behcet disease [44] are also associated with elevated NLR values. Moreover, NLR correlates with coronary and thoracic periaortic calcification in ESRD [1] and is an independent factor for increased carotid-femoral pulse wave velocity (cfPWV), reflecting arterial stiffness, as well as cardiovascular mortality in patients undergoing peritoneal dialysis (PD) [19].

NLR level ≥3.5 among HD patients [16] and ≥4.5 in general population [19] is associated with all-cause and cardiovascular mortality, which is in agreement with our examination. Thus, the NLR may be the new, easily obtained, and promising indicator of inflammation and can identify the high risk of cardiovascular diseases [16] and mortality in chronic HD patients early on [3, 16, 18]. The most common inflammatory marker, which is CRP, is not as precise as NLR [39]. Additionally, Dawood et al. did not find the correlation between CRP and the QRS-T angle [48], similarly as in our paper. It seems that NLR and CRP may determine various pathways of inflammatory condition, which is in accordance with results of Kwon et al. [15]. ROC analysis in our study showed that NLR greater than 4.59 is the best cut-off point value for an increased QRS-T angle and it is in agreement with previous studies by Durmus et al. [49] and Vano et al. [50]. Despite the fact that its pathogenesis is not fully known and needs further examination, NLR is thought to be a better indicator of myocardial inhomogeneities reflected by the wide spatial QRS-T angle than CRP, which is also consistent with other studies [16, 29, 39].

Our study has revealed that NLR is an inexpensive, easily obtained, and precise biomarker of inflammation reflecting myocardial inhomogeneities assessed as a widened QRS-T angle.

## 5. Limitations

This study has some limitations. Firstly, the group of patients was relatively small; nevertheless, it was big enough to conduct the above analysis. Secondly, among inflammation markers that may affect the QRS-T angle, only NLR and CRP were measured. The third limitation of this study was that certain parameters were marked only once. The assessment of NLR over time could probably positively affect its usefulness in QRS-T angle prediction in HD patients.

## 6. Conclusion

Inflammation may play a role in pathogenesis of myocardial inhomogeneities in HD patients. Thus, NLR as marker of inflammation and the independent predictor of spatial QRS-T angle might indicate this clinical state in the analyzed group of patients.

## Figures and Tables

**Table 1:** Basic biochemical parameters of the examined patients.

Parameter	Mean (*X*) ± standard deviation (SD) or median (*M*) (minimum–maximum)
Age (years)	64.96 ± 10.92
Hemoglobin (g/dl)	10.45 ± 1.58
Hematocrit (%)	31.71 ± 5.04
Neutrophils (×10^3^/*μ*l)	4.18 ± 1.72
Lymphocytes (×10^3^/*μ*l)	1.49 ± 0.53
Neutrophil-lymphocyte ratio	3.40 ± 1.29
C-reactive protein (mg/l)	8.35 (0.11-86.9)
Parathormone (pg/ml)	392.04 (8-1235)
Calcium (md/dl)	4.09 ± 0.36
Potassium (mEq/l)	5.12 ± 1.00
Ferritin (*μ*g/l)	867.79 ± 482.04
Cholesterol (mg/dl)	188.3 ± 37.35
LDL cholesterol (mg/dl)	114.8 ± 30.11
HDL cholesterol (mg/dl)	43.82 ± 17.08
Triglycerides (mg/dl)	176.3 ± 59.27

Data are expressed as the mean ± SD, except for PTH and CRP, which are expressed as the median and ranges.

**Table 2:** Correlations of selected parameters with spatial QRS-T angle.

Parameter	*r*	*p* value
Left ventricle mass index	0.61	<0.001
Left atrial volume index	0.46	<0.001
E/E`	0.61	<0.001
Left ventricle ejection fraction	-0.675	<0.001
QRS duration	0.431	<0.001
QTc	0.54	<0.001
Neutrophil-lymphocyte ratio	0.498	<0.001
Parathormone	-0.201	0.021
Calcium	-0.349	<0.001
C-reactive protein	0.411	<0.001

**Table 3:** Detailed analysis of multiple regression analysis describing the dependence between QRS-T angle and parameters selected based on Pearson's correlations presented in Table 2.

*N* = 183	*R* = 0.807; *R*^2^ = 0.651; corrected *R*^2^ = 0.633; *F*(7.136) = 36.289; *p* < 0.0001					
	*b*∗	SD of *b*∗	*b*	SD of *b*	*t*	*p*
			136.142	24.211	5.623	<0.001
Left ventricle mass index (LVMI)	0.505	0.084	1.174	0.196	5.977	<0.001
Left atrial volume index (LAVI)	0.034	0.071	0.033	0.068	0.478	0.634
Left ventricle ejection fraction (EF)	-0.355	0.078	-0.861	0.189	-4.562	<0.001
Parathormone (PTH)	0.046	0.068	0.002	0.003	0.683	0.500
Neutrophil-lymphocyte ratio	-0.159	0.052	-2.646	0.865	-3.060	0.003
Calcium (Ca)	-0.053	0.068	-3.226	4.086	-0.790	0.431

SD: standard deviation.

**Table 4:** The detailed description of ROC curve analysis.

	Area under curve	95% confidence interval (CI)	Sensitivity/specificity	Cut-off point
Neutrophil-lymphocyte ratio (NLR)	0.601	0.569-0.662	0.677/0.601	4.59

## Data Availability

All data used and/or analyzed in the present study are presented in the manuscript or available from the corresponding author on request.
